# Formation of Oxidatively Modified Lipids as the Basis for a Cellular Epilipidome

**DOI:** 10.3389/fendo.2020.602771

**Published:** 2020-12-21

**Authors:** Corinne M. Spickett

**Affiliations:** School of Biosciences, College of Health and Life Sciences, Aston University, Aston Triangle, Birmingham, United Kingdom

**Keywords:** phospholipids (PL), oxidation, nitration, oxysterols (cholesterol oxidation products), free radicals, hypochlorous acid (HOCl)

## Abstract

While often regarded as a subset of metabolomics, lipidomics can better be considered as a field in its own right. While the total number of lipid species in biology may not exceed the number of metabolites, they can be modified chemically and biochemically leading to an enormous diversity of derivatives, many of which retain the lipophilic properties of lipids and thus expand the lipidome greatly. Oxidative modification by radical oxygen species, either enzymatically or chemically, is one of the major mechanisms involved, although attack by non-radical oxidants also occurs. The modified lipids typically contain more oxygens in the form of hydroxyl, epoxide, carbonyl and carboxylic acid groups, and nitration, nitrosylation, halogenation or sulfation can also occur. This article provides a succinct overview of the types of species formed, the reactive compounds involved and the specific molecular sites that they react with, and the biochemical or chemical mechanisms involved. In many cases, these modifications reduce the stability of the lipid, and breakdown products are formed, which themselves have interesting properties such as the ability to react with other biomolecules. Publications on the biological effects of modified lipids are growing rapidly, supporting the concept that some of these biomolecules have potential signaling and regulatory effects. The question therefore arises whether modified lipids represent an “epilipidome”, analogous to the epigenetic modifications that can control gene expression.

## Introduction

**Figure 1:**
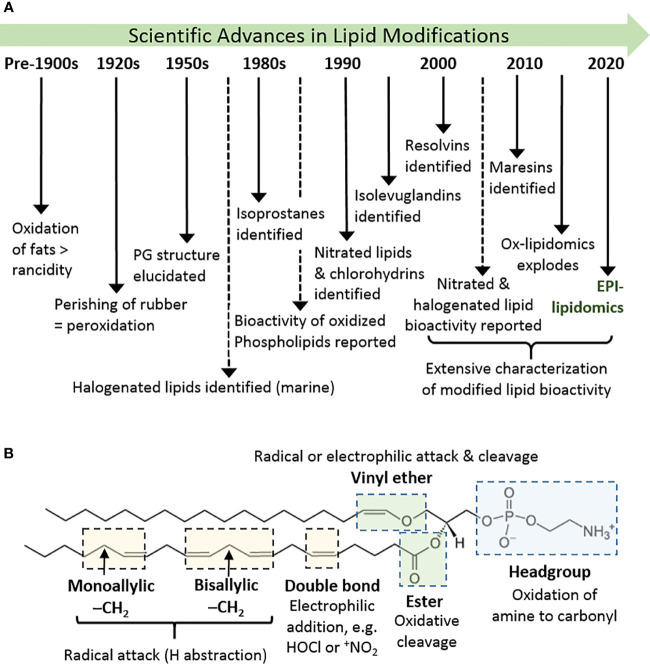
History and basics of lipid oxidation. **(A)** Diagrammatic timeline of research into lipid oxidation identifying key discoveries and concepts. **(B)** Major site sites of attack in phospholipids and types of reaction that can occur there, using 1-(1-octadecenyl)-2-arachidonoyl-sn-glycero-3-phosphoethanolamine as an example. Other phospholipids containing these or analogous chemical groups show similar susceptibility.

The oxidation of lipids and lipid-like substances has been known for centuries, and has been widely regarded as an undesirable effect: in foods, lipid oxidation leads to the development of rancidity and acrid flavors, while in materials such as rubber it causes loss of elasticity and perishing (1). In biology, where lipids have important structural, nutritional, and signaling roles, the adventitious, radical oxidation of lipids in cells and tissues was for many years also be regarded as a detrimental process, for example disrupting cell membranes and causing cytotoxicity (**Figure 1A**) (2, 3). On the other hand, in the 1950s the structure of prostaglandins was elucidated and found to result from peroxidation of arachidonic acid [reviewed by (4)]; subsequently, thromboxanes and leukotrienes were also realized to be derived from hydroperoxyeicosatetraenoates (HPETEs) (5). These enzymatically generated non-esterified lipid products were recognized as important signalling molecules in the cardiovascular and immune systems, and therefore as important therapeutic targets (6). Consequently, there was much interest in their enzymatic production by cyclooxygenases, lipoxygenases, and cytochrome P450-dependent enzymes (7), a topic that continues to be of interest and is reviewed elsewhere in this issue. Later, the non-enzymatic formation of analogous compounds (F_2_-isoprostanes) was discovered (8) and, in parallel, evidence began to emerge that non-enzymatic oxidation products of fatty acids esterified in phospholipids also had biological activities (9). While initial studies reported detrimental effects in atherosclerosis, soon it was noted that some of these compounds were able to block immune receptors and prevent damaging immune responses, e.g. in sepsis (10). The years from 2000 onwards witnessed an explosion in the identification of non-enzymatic lipid modifications and resulting biological effects. A wide variety of additional oxidation product families were identified, including isolevuglandins, nitrated and halogenated fatty acids or phospholipids, oxysterols and halogenated sterols, as well as the discovery of resolvins (11) and maresins (12) from oxidation products of the omega-3 fatty acids eicosapentaenoic acid (EPA), docosapentaenoic acid (DPA) and docosahexaenoic acid (DHA). In most cases, a strong driver in their discovery has been the elucidation of biological signalling effects and, as the field has evolved, it has become clear that certain modified lipid species have beneficial effects in specific circumstances; in many cases, we also have an understanding of the mechanisms involved. Thus, oxidatively modified lipids are now well-established as mediators of biological processes (2, 13–16).

## Chemical Properties of Lipids That Enable Modifications

Lipids are a hugely diverse chemical group, but the lipid species most prevalent in biological systems, and especially in mammalian cells, are free fatty acids, ceramides, phospholipids (including phosphatidylglycerols and sphingomyelin), mono-, di- and tri-acylglycerols, and sterols. The lipid structure determines the nature and likelihood of oxidative modifications to it, but reactive oxidizing compounds also demonstrate different specificities (17). The chemical moieties most typically susceptible to oxidative attack and modifications are shown in **Figure 1B**. In general, these are electron-dense regions of the molecules (double bonds), or ones where the bonds are polarized and can be broken with lower energy input.

The site of attack that leads to the widest range of modifications and oxidation products is the fatty acyl chain. Although fully saturated hydrocarbon chains can be attacked by high energy oxidants, e.g. ozone and triplet oxygen, higher numbers of double bonds increase the susceptibility to radical attack, as hydrogen atoms can more easily be abstracted from bis-allylic carbon atoms (17). On the other hand, mono-unsaturated fatty acyl chains react readily with non-radical oxidants, such as hypochlorous acid (18). In sphingomyelins, the sphingosine moiety appears to be the main site of modification, at least by hydroxyl radicals, reflecting the presence of a C-C double bond (19). Likewise, in cholesterol the mono-unsaturated B ring is readily oxidized, although enzymatic oxidation of the tail also occurs (20–22).

In phospholipids, fatty acyl chains are connected to the glycerol backbone by 3 different types of bond: ester bonds, ether bonds (in alkanyl phospholipids), or vinyl ether bonds (in alkenyl phospholipids, also called plasmalogens). The ether or vinyl ether bonds occur mostly commonly at the SN-1 position of the glycerol. The ester bonds are most common biologically and can be hydrolyzed enzymatically, for example by phospholipase A_1_ or A_2_, which results in formation of lysophospholipids. These have altered biological properties and can be considered as biological mediators. In contrast, vinyl ether bonds are susceptible to attack by radicals (23) and electrophilic oxidants (24), forming oxidant-dependent products. Phospholipid headgroups containing an amine group can also undergo oxidation, although the quaternary ammonium structure of phosphocholine is resistant; changes in headgroup structure are likely to impact significantly on the phospholipid function within the cell membrane (25, 26).

## Types of Lipid Modifications

**Figure 2:**
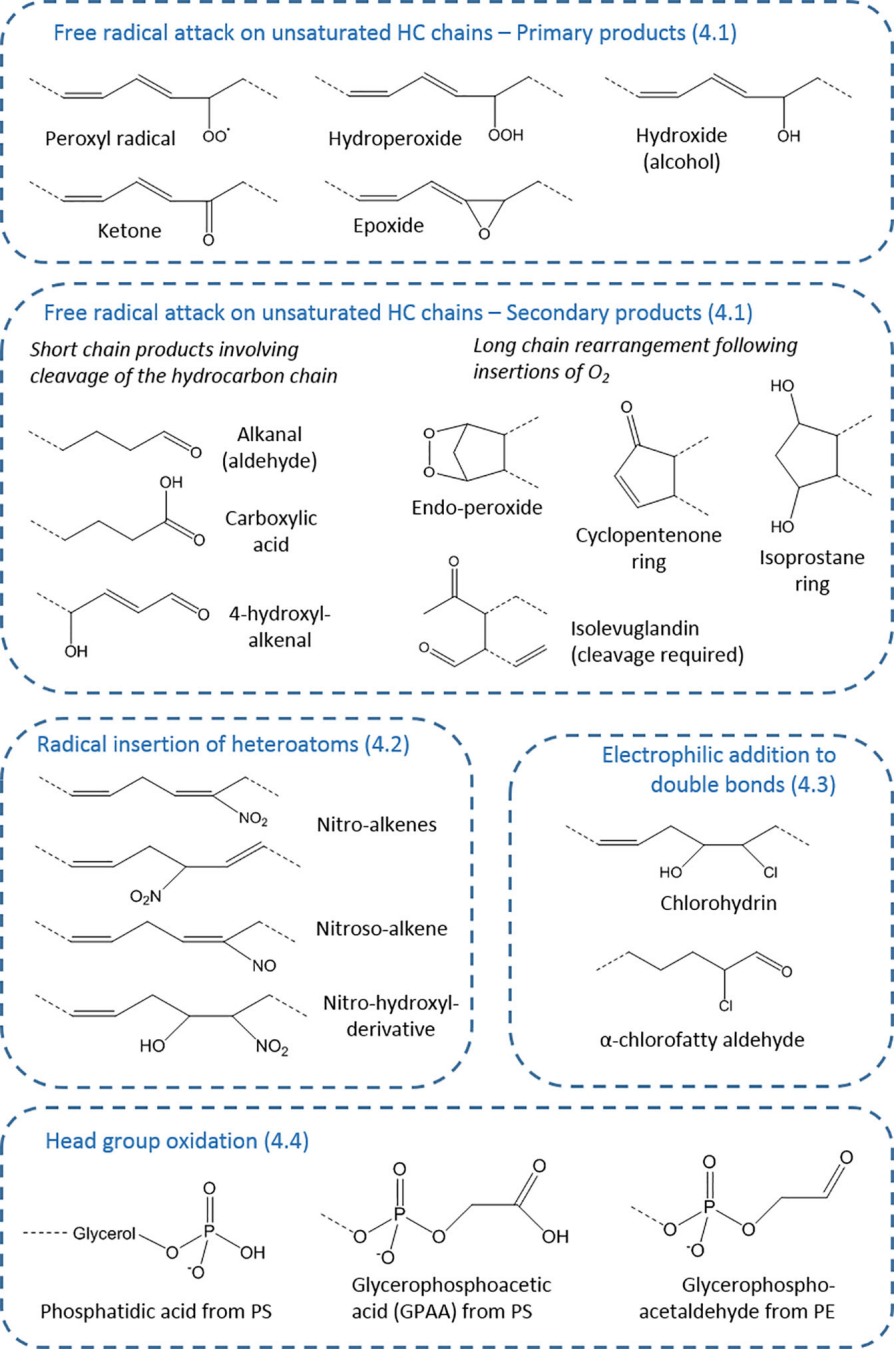
Types of oxidative modifications on fatty acyl chains. The products are organized according to section of the article (numbered), showing the wide variety of chemical structures possible. These chemical moieties can occur on esterified or non-esterified fatty acyl chains, or cholesterol, and for each generic structure many distinct compounds (isomers and stereoisomers) may exist—for example, 64 in the case of isoprostanes—as well as analogous compounds from starting lipids with different chain length and unsaturation.

The variety of sites of modification in lipids present the basis for the large range of products that can be formed (27), but this is expanded by the type of oxidant that causes the modification and the stability or otherwise of the initial product. This aspect will be explored in the following sections to illustrate the potential for diversity in modified lipids. **Figure 2** provides an overview of the key types of products.

### Peroxidation of Fatty Acyl Chains Caused by Free Radical Attack

Whether enzymatic or non-enzymatic lipid modification is considered, radical attack leads to the widest range of products, largely because of the unstable nature of the initial oxidation products, their potential for rearrangement, and subsequent breakdown or fragmentation. For hydrocarbon chains, radical attack involves the abstraction of a hydrogen to form a carbon-centered radical, and leads to formation of a peroxide by incorporation of molecular oxygen (28). The potential for rearrangement at carbon radical stage depends on the degree and nature of unsaturation in the local area; for example, whether it is a conjugated system.

Hydrogen abstraction at bis-allylic carbons is favored, although it can also occur at allylic sites. This makes polyunsaturated fatty acyl chains such as linoleate (1 bis-allylic carbon); linolenate acid (2 bis-allylic carbons), arachidonate (3 bis-allylic carbons), eicosapentenoate (4 bis-allylic carbons), and docosahexenoate (5 bis-allylic carbons) increasingly susceptible to peroxidation, which can occur at multiple sites (3, 17). As the extent of modification by oxygen increases, the complexity of the oxidation product set increases, and their stability decreases. The initial product is a peroxyl radical, which can either react intramolecularly to form an endoperoxide in which the molecule retains an unpaired electron, or it can abstract a hydrogen from an adjacent molecule to form a hydroperoxide, concomitantly initiating the chain reaction of lipid peroxidation.

Endoperoxide formation is central in the formation of a number of bioactive oxidized lipid families, including the isoprostanes (29). Rearrangement of the endoperoxide results in formation of 5-membered ring structures, such as cyclopentenone rings, which are present in isoprostanes and their enzymatic analogues prostaglandins (30). Alternatively, the hydrocarbon chain can be cleaved to form the highly reactive compounds isolevuglandins, which are di-aldehydes (31, 32). Similar reactions also result in formation of the lipid oxidation breakdown product malondialdehyde.

In contrast, the hydroperoxides are relatively stable, and can be detected in biological samples following organic extraction and storage at -20°C or lower (33). Hydroperoxides can be reduced through the action of phospholipid-dependent glutathione peroxidase (GPx4), which converts the hydroperoxide to an alcohol (34, 35), although a mechanism for removing the –OH moiety to regenerate the hydrocarbon chain is not currently known. Hydroperoxides can also be converted to epoxides through homolytic cleavage of the hydroperoxide to form an alkoxyl radical, which attacks the adjacent carbon atom (36).

Either peroxyl radical, endoperoxides, or hydroperoxides can undergo intra-molecular reactions leading to the fragmentation of the carbon chain, which usually generates an aldehyde at one or both sides of the cleavage site. This process is responsible for the formation of a variety of lipid peroxidation breakdown products, of which the best-known example is 4-hydroxynonenal, in parallel with the corresponding chain-shortened phospholipid (37). These products can subsequently be metabolized by enzymes of the aldoketoreductase (AKR) and aldehyde dehydrogenase families, involving either reduction to alcohols or oxidation to carboxylic acids (38–40), thus generating further product diversity. An idea of the extent of the possible diversity can be obtained by considering that addition of two molecular oxygens to arachidonate can yield a family of 64 F_2_-isoprostanes, when stereoisomers are included (41). Moreover, fragmentation of oxidized phospholipids can yield multiple breakdown products, and analysis is challenging as ones from different parent lipids may be isomeric or isobaric, as observed by liquid chromatography tandem mass spectrometry (42).

Analogous reactions can also take place on cholesterol and sphingolipid chains. Radical oxidation of cholesterol yields a family of oxysterols modified on the B-ring, including 7-hydroxycholesterol, 7-keto cholesterol, 5-hydroperoxycholesterol, 5,6-epoxycholesterol and cholestane-3,5,6- triol (43). In contrast, enzymatic oxidation catalyzed by cytochrome P450 enzymes (e.g. CYP27A1, CYP46A1) tends to hydroxylate the saturated hydrocarbon tail, although 7a-hydroxycholesterol is formed by CYP7A1 (20). Carotenoids contain conjugated polyunsaturated chains and are also highly susceptible to oxidative attack; carotene oxidation products reported include cyclized hydroxy- and keto-containing as well as aldehydes resulting from chain cleavage (44, 45). Oxidation of sphingosylphosphorylcholine has been observed to form hydroxyl and keto derivatives on the sphingosine chain (19).

The ability of radicals to initiate hydrogen abstraction varies. Hydroxyl radical (OH^•^) is one of the most reactive radicals formed in biological systems, and readily causes lipid peroxidation (46). In contrast, superoxide, a radical produced by certain NADPH oxidases, is relatively poor at initiating lipid peroxidation, and hydrogen peroxide is unable to do this in the absence of transition metal ions that support Fenton chemistry to generate hydroxyl radicals (17); transition metals such as copper, manganese and iron readily undergo one-electron (radical) reactions. Similarly, the non-radical anion peroxynitrite (ONOO^-^) does not cause hydrogen abstraction directly, although it is reactive and can be converted to nitrogen-containing radicals such as nitrogen dioxide that do, and also reacts with carbon dioxide to form carbonate radicals $\left(CO_{3}^{-\cdot }\right)$ that enhance peroxidation (47). Although a radical, nitric oxide (NO) is a better reductant than oxidant in biological systems (48). Radical nitrogen species can also be generated by the neutrophil enzyme myeloperoxidase; as well as its conventional non-radical product hypochlorous acid, it is able to oxidize nitric oxide to form the radical NO_2_, and can also oxidize other compounds, for example tyrosine to yield tyrosyl radicals (49, 50). Oxygen itself is a di-radical and can initiate the direct peroxidation of dry lipid monolayers *in vitro* (auto-oxidation), but this process may not be biologically relevant as the oxygen concentration in cell membranes is much lower than air.

### Modification of Fatty Acyl Chains by Radical Nitrogen Species

As well causing peroxidation, reactive nitrogen radicals can also cause nitration and nitroxidation of unsaturated fatty acyl chains, and the resulting nitrated lipids have important biological functions, for example as anti-inflammatory agents and stress signaling molecules in both animals and plants (51–53). The formation of nitrogen-containing oxidized lipid derivatives was first documented in the mid-1990s (54) and was rapidly followed by further mechanistic studies of nitration reactions (55). Radical-initiated nitration can occur by two distinct mechanisms. The first requires hydrogen abstraction by a radical followed by addition of NO_2_ at the carbon-centred radical, in a mechanism analogous to lipid peroxidation. Under acidic conditions, peroxynitrite is converted to peroxynitrous acid (ONOOH), which decomposes to form OH^•^ and NO_2_; thus hydroxyl radical initiates the hydrogen abstraction followed by addition of NO_2_ to nitrate the hydrocarbon chain, forming a nitro-lipid (51, 56). The radical NO^•^ could also undergo a radical condensation with the carbon-centred radical, which would result in lipid nitrosylation. NO_2_ can also react directly with one of the carbons in the double bond to form a nitroalkane radical, and if the NO_2_ concentration is high a second nitration can occur to yield a di-nitro species. Subsequent loss of nitrous acid (HNO_2_) leads to nitro-alkenes, and substitution with water can form nitrohydroxy lipids (51). As with oxidation products resulting from free radical attack, the molecular rearrangements of nitro-lipids allow a wide variety of positional and stereochemical isomers to be formed, for example on phosphatidylserine (57), cardiolipin (58), phosphatidylcholine, and phosphatidylethanolamine (59). Nitrated fatty acids have been detected in human plasma, suggesting that they are biologically relevant lipid products (60). Nitration of unsaturated fatty acids can also occur by non-radical electrophilic substitutions, as described in the following section.

### Electrophilic Attack by Non-Radical Species

Unsaturated fatty acids and fatty acyl chains of phospholipids can be oxidatively modified in a non-radical manner *via* electrophilic addition of oxidants to double bonds. For example, addition of the reactive nitronium ion $\left(NO_{2}^{+}\right)$, usually from a polarized nitronium carrier such as nitronium hexafluorophosphate, generates nitroalkenes (51), although it is not clear that such a mechanism is biologically relevant. In contrast, electrophilic addition of hypohalous acids to unsaturated lipids is better established, with more evidence for its occurrence *in vivo*. Hypohalous acids include hypochlorous acid (HOCl), hypobromous acid (HOBr), and hypoiodous acid (HOI) and are produced mainly by phagocytes (18). The main source of HOCl is the neutrophil enzyme myeloperoxidase; this enzyme has a higher Km for bromide than chloride, but the higher biological chloride levels mean that HOCl is the major product (61, 62). Eosinophil peroxidase is a related enzyme that is highly selective for HOBr production (61).

Hypohalous acids can add across double bonds in unsaturated fatty acyl chains to form halohydrins: the products on mono-unsaturated chains (e.g. mono-chlorohydrins) are fairly stable, but reaction with poly-unsaturated chains leads to a large number of products through rearrangement by loss of water or loss of chlorine, with the possibility of further reactions in the presence of high concentrations of HOCl (7). Chlorohydrins of fatty acids (adjacent hydroxy and chloro groups) have been detected in clinical conditions such as acute pancreatitis and sepsis (63, 64). Hypohalites can also attack vinyl ether bonds in plasmalogen phosholipids, which causes cleavage to form a lysophospholipid and releases an α-halo-fatty aldehyde (24, 65). This contrasts with radical attack of plasmalogens, which yields fatty aldehydes (23). It has most commonly been reported for HOCl, and α-chloro hexadecanal and α-chloro octadecanal have been detected in plasma of patients with cardiovascular disease (66) and sepsis (67, 68), but bromo-fatty aldehydes can also be formed (69). HOCl can react with the double bond in cholesterol to form 5-chloro-6-hydroxy-cholesterol and its isomer (70); these were reported in cell membranes (71) and subsequently myeloperoxidase-derived chlorine was reported to form a family of chlorinated sterols (72). HOCl can react with β-carotene and shows overlap in the products formed by free radical cleavage (73). Thus although the variety of halogenated products is less than that from radical oxidation, it still adds substantially to the modified lipid family.

### Modifications of Phospholipid Headgroups

Although attention tends to focus on hydrocarbon chain oxidation, amine-containing phospholipid head groups can be attacked both by radicals and electrophilic oxidants. The photooxidation of phosphatidylethanolamines (PE) has been demonstrated to cause loss of ethanolamine to form phosphatidic acid; interestingly, glycation by reaction with the amine enhanced the propensity for oxidation and led to oxidative cleavages in the glucose unit (**Figure 2**) (74, 75). The ethanolamine head group can also be modified by reaction with isolevuglandins (76), illustrating the complexity of effects of phospholipid oxidation, and such products have been detected in cells (77). Radical oxidation of phosphatidylserine (PS) typically yields glycero-3-phosphoacetic acid (GPAA) *via* oxidative deamination (78, 79), whereas glycero-3-phosphoacetaldehyde and glycero-3-phosphonitrile were observed following reaction with HOCl (80). These modifications are important as the head groups play key roles in membrane structure and function, as well as cell signaling.

## Discussion

It is clear that oxidative modifications of lipids are legion, resulting a substantial expansion in the variety and properties of lipids. Many of the oxidized, nitrated, and chlorinated products show altered biological activities, including toxicity, altered proliferation, differentiation, pro-inflammatory, anti-inflammatory and barrier protective effects, *via* diverse signalling pathways to affect gene expression or other regulatory processes. In this sense, the modifications offer a chemical/biochemical mechanism to alter cell behaviour in both beneficial and deleterious ways, and to some extent meet the concept of an epilipidome. There is a close analogy to the recent shift in thinking on “reactive oxygen species (ROS)” as potentially beneficial signalling compounds, rather than agents of destruction (81, 82). On the other hand, the modifications underlying epigenetics are reversible and enzyme-catalyzed, offering clear evidence that they are a regulatory process. The recent concept of epi-proteomics also depends on the principle of reversibility: many post-translational modifications are enzymatically controlled and reversible, e.g. phosphorylation, and histone acetylation (83, 84). In contrast, the same cannot be said of lipid oxidation. While some enzymes are specific for lipid oxidation products, such as GPx4, aldoketo reductases and aldehydes dehydrogenases, these constitute metabolism rather than direct reversibility. On this basis, the epilipidome would function in the sense of a metabolic loop, involving formation and degradation *via* distinct pathways. It is also worth bearing in mind that reactive lipid oxidation products exert at least some of their effects *via* covalent interactions with proteins in the form of post-translational modification known as lipoxidation, and these reactions are chemically reversible (85). In view of the wide variety of cellular effects reported for modified lipids, as well as its role in ferroptosis (86, 87) and inflammatory diseases (88), it is important to continue to explore their potential as an epilipidome, including aspects of reversibility and enzyme interaction. This will require development of new technologies to handle the large datasets of modified lipids that form the epilipidome (89).

## Author Contributions

CMS is the sole author and therefore responsible for all aspects of this article.

## Conflict of Interest

The author declares that the research was conducted in the absence of any commercial or financial relationships that could be construed as a potential conflict of interest.

## References

[1] Hammond EG White PJ A Brief History of Lipid Oxidation. J Am Oil Chem Soc (2011) 88(7):891–7. 10.1007/s11746-011-1761-8

[2] Spickett CM Chapter 15 - Oxidized phospholipid signaling: Distress to eustress. In: Sies H, editor. Oxidative Stress. Oxford, UK: Academic Press (2020). p. 263–85.

[3] Catala A A synopsis of the process of lipid peroxidation since the discovery of the essential fatty acids. Biochem Biophys Res Commun (2010) 399(3):318–23. 10.1016/j.bbrc.2010.07.087 20674543

[4] Montuschi P Barnes P Roberts LJ,2 Insights into oxidative stress: the isoprostanes. Curr Med Chem (2007) 14(6):703–17. 10.2174/092986707780059607 17346157

[5] Samuelsson B An elucidation of the arachidonic acid cascade. Discovery of prostaglandins, thromboxane and leukotrienes. Drugs (1987) 33(Suppl 1):2–9. 10.2165/00003495-198700331-00003 3036460

[6] Kuehl FA Jr Egan RW Prostaglandins, arachidonic acid, and inflammation. Science (1980) 210(4473):978–84. 10.1126/science.6254151 6254151

[7] Spickett CM Fauzi NM Analysis of oxidized and chlorinated lipids by mass spectrometry and relevance to signalling. Biochem Soc Trans (2011) 39(5):1233–9. 10.1042/BST0391233 21936795

[8] Morrow JD Hill KE Burk RF Nammour TM Badr KF Roberts LJ,2 A series of prostaglandin F2-like compounds are produced in vivo in humans by a non-cyclooxygenase, free radical-catalyzed mechanism. Proc Natl Acad Sci U S A (1990) 87(23):9383–7. 10.1073/pnas.87.23.9383 PMC55169 2123555

[9] Watson AD Leitinger N Navab M Faull KF Horkko S Witztum JL Structural identification by mass spectrometry of oxidized phospholipids in minimally oxidized low density lipoprotein that induce monocyte/endothelial interactions and evidence for their presence in vivo. J Biol Chem (1997) 272(21):13597–607. 10.1074/jbc.272.21.13597 9153208

[10] Bochkov VN Kadl A Huber J Gruber F Binder BR Leitinger N Protective role of phospholipid oxidation products in endotoxin-induced tissue damage. Nature (2002) 419(6902):77–81. 10.1038/nature01023 12214235

[11] Serhan CN Hong S Gronert K Colgan SP Devchand PR Mirick G Resolvins: a family of bioactive products of omega-3 fatty acid transformation circuits initiated by aspirin treatment that counter proinflammation signals. J Exp Med (2002) 196(8):1025–37. 10.1084/jem.20020760 PMC2194036 12391014

[12] Serhan CN Yang R Martinod K Kasuga K Pillai PS Porter TF Maresins: novel macrophage mediators with potent antiinflammatory and proresolving actions. J Exp Med (2009) 206(1):15–23. 10.1084/jem.20081880 19103881 PMC2626672

[13] Karki P Birukov KG Oxidized Phospholipids in Healthy and Diseased Lung Endothelium. Cells (2020) 9(4):981. 10.3390/cells9040981 PMC7226969 32326516

[14] O’Donnell VB Aldrovandi M Murphy RC Kronke G Enzymatically oxidized phospholipids assume center stage as essential regulators of innate immunity and cell death. Sci Signal (2019) 12(574):eaau2293. 10.1126/scisignal.aau2293 30914483

[15] Bochkov V Gesslbauer B Mauerhofer C Philippova M Erne P Oskolkova OV Pleiotropic effects of oxidized phospholipids. Free Radic Biol Med (2017) 111:6–24. 10.1016/j.freeradbiomed.2016.12.034 28027924

[16] Greig FH Kennedy S Spickett CM Physiological effects of oxidized phospholipids and their cellular signaling mechanisms in inflammation. Free Radic Biol Med (2012) 52(2):266–80. 10.1016/j.freeradbiomed.2011.10.481 S0891-5849(11)01158-0 [pii]. 22080084

[17] Reis A Spickett CM Chemistry of phospholipid oxidation. Biochim Biophys Acta (2012) 1818(10):2374–87. 10.1016/j.bbamem.2012.02.002 22342938

[18] Spickett CM Chlorinated lipids and fatty acids: an emerging role in pathology. Pharmacol Ther (2007) 115(3):400–9. 10.1016/j.pharmthera.2007.06.002 17658610

[19] Melo T Maciel E Oliveira MM Domingues P Domingues MRM Study of sphingolipids oxidation by ESI tandem MS. Eur J Lipid Sci Technol (2012) 114(7):726–32. 10.1002/ejlt.201100328

[20] Olkkonen VM Beaslas O Nissila E Oxysterols and their cellular effectors. Biomolecules (2012) 2(1):76–103. 10.3390/biom2010076 24970128 PMC4030866

[21] Niki E Oxidant-specific biomarkers of oxidative stress. Association with atherosclerosis and implication for antioxidant effects. Free Radic Biol Med (2018) 120:425–40. 10.1016/j.freeradbiomed.2018.04.001 29625172

[22] Vurusaner B Leonarduzzi G Gamba P Poli G Basaga H Oxysterols and mechanisms of survival signaling. Mol Aspects Med (2016) 49:8–22. 10.1016/j.mam.2016.02.004 27017897

[23] Khaselev N Murphy RC Structural characterization of oxidized phospholipid products derived from arachidonate-containing plasmenyl glycerophosphocholine. J Lipid Res (2000) 41(4):564–72. 10744777

[24] Palladino END Hartman CL Albert CJ Ford DA The chlorinated lipidome originating from myeloperoxidase-derived HOCl targeting plasmalogens: Metabolism, clearance, and biological properties. Arch Biochem Biophys (2018) 641:31–8. 10.1016/j.abb.2018.01.010 PMC5817035 29378164

[25] Yusupov M Wende K Kupsch S Neyts EC Reuter S Bogaerts A Effect of head group and lipid tail oxidation in the cell membrane revealed through integrated simulations and experiments. Sci Rep (2017) 7(1):5761. 10.1038/s41598-017-06412-8 28720839 PMC5515852

[26] Madrid E Horswell SL Effect of headgroup on the physicochemical properties of phospholipid bilayers in electric fields: size matters. Langmuir (2013) 29(5):1695–708. 10.1021/la304455d 23331178

[27] Davies SS Guo L Lipid Peroxidation and Nitration. In: Villamena FA, editor. Molecular Basis of Oxidative Stress . John Wiley & Sons (2013). p. 49–70.

[28] Spickett CM Wiswedel I Siems W Zarkovic K Zarkovic N Advances in methods for the determination of biologically relevant lipid peroxidation products. Free Radic Res (2010) 44(10):1172–202. 10.3109/10715762.2010.498476 20836661

[29] Yin H Havrilla CM Gao L Morrow JD Porter NA Mechanisms for the formation of isoprostane endoperoxides from arachidonic acid. “Dioxetane” intermediate versus beta-fragmentation of peroxyl radicals. J Biol Chem (2003) 278(19):16720–5. 10.1074/jbc.M300604200 12609993

[30] Fam SS Murphey LJ Terry ES Zackert WE Chen Y Gao L Formation of highly reactive A-ring and J-ring isoprostane-like compounds (A4/J4-neuroprostanes) in vivo from docosahexaenoic acid. J Biol Chem (2002) 277(39):36076–84. 10.1074/jbc.M205638200 12133837

[31] Salomon RG Batyreva E Kaur K Sprecher DL Schreiber MJ Crabb JW Isolevuglandin-protein adducts in humans: products of free radical-induced lipid oxidation through the isoprostane pathway. Biochim Biophys Acta (2000) 1485(2–3):225–35. 10.1016/s1388-1981(00)00038-x 10832102

[32] Zhang M Li W Li T Generation and detection of levuglandins and isolevuglandins in vitro and in vivo. Molecules (2011) 16(7):5333–48. 10.3390/molecules16075333 PMC6264246 21705973

[33] Spickett CM Rennie N Winter H Zambonin L Landi L Jerlich A Detection of phospholipid oxidation in oxidatively stressed cells by reversed-phase HPLC coupled with positive-ionization electrospray [correction of electroscopy] MS. Biochem J (2001) 355(Pt 2):449–57. 10.1042/0264-6021:3550449 PMC1221757 11284733

[34] Cozza G Rossetto M Bosello-Travain V Maiorino M Roveri A Toppo S Glutathione peroxidase 4-catalyzed reduction of lipid hydroperoxides in membranes: The polar head of membrane phospholipids binds the enzyme and addresses the fatty acid hydroperoxide group toward the redox center. Free Radic Biol Med (2017) 112:1–11. 10.1016/j.freeradbiomed.2017.07.010 28709976

[35] Ursini F Maiorino M Roveri A Phospholipid hydroperoxide glutathione peroxidase (PHGPx): more than an antioxidant enzyme? BioMed Environ Sci (1997) 10(2-3):327–32. 9315326

[36] Schneider C Boeglin WE Yin H Porter NA Brash AR Intermolecular peroxyl radical reactions during autoxidation of hydroxy and hydroperoxy arachidonic acids generate a novel series of epoxidized products. Chem Res Toxicol (2008) 21(4):895–903. 10.1021/tx700357u 18324788

[37] Spickett CM The lipid peroxidation product 4-hydroxy-2-nonenal: Advances in chemistry and analysis. Redox Biol (2013) 1:145–52. 10.1016/j.redox.2013.01.007 PMC3757682 24024147

[38] Singh M Kapoor A Bhatnagar A Oxidative and reductive metabolism of lipid-peroxidation derived carbonyls. Chem Biol Interact (2015) 234:261–73. 10.1016/j.cbi.2014.12.028 PMC4414726 25559856

[39] Spite M Baba SP Ahmed Y Barski OA Nijhawan K Petrash JM Substrate specificity and catalytic efficiency of aldo-keto reductases with phospholipid aldehydes. Biochem J (2007) 405(1):95–105. 10.1042/BJ20061743 17381426 PMC1925154

[40] Li D Ferrari M Ellis EM Human aldo-keto reductase AKR7A2 protects against the cytotoxicity and mutagenicity of reactive aldehydes and lowers intracellular reactive oxygen species in hamster V79-4 cells. Chem Biol Interact (2012) 195(1):25–34. 10.1016/j.cbi.2011.09.007 22001351

[41] Taber DF Morrow JD Roberts LJ,2 A nomenclature system for the isoprostanes. Prostaglandins (1997) 53(2):63–7. 10.1016/s0090-6980(97)00005-1 9112285

[42] Gruber F Bicker W Oskolkova OV Tschachler E Bochkov VN A simplified procedure for semi-targeted lipidomic analysis of oxidized phosphatidylcholines induced by UVA irradiation. J Lipid Res (2012) 53(6):1232–42. 10.1194/jlr.D025270 PMC3351830 22414483

[43] Zerbinati C Iuliano L Cholesterol and related sterols autoxidation. Free Radic Biol Med (2017) 111:151–5. 10.1016/j.freeradbiomed.2017.04.013 28428001

[44] Schieber A Weber F 5 - Carotenoids. In: Carle R Schweiggert RM, editors. Handbook on Natural Pigments in Food and Beverages. Cambridge, UK: Woodhead Publishing (2016). p. 101–23.

[45] Siems W Salerno C Crifo C Sommerburg O Wiswedel I Beta-carotene degradation products - formation, toxicity and prevention of toxicity. Forum Nutr (2009) 61:75–86. 10.1159/000212740 19367112

[46] Phaniendra A Jestadi DB Periyasamy L Free radicals: properties, sources, targets, and their implication in various diseases. Indian J Clin Biochem (2015) 30(1):11–26. 10.1007/s12291-014-0446-0 25646037 PMC4310837

[47] Carballal S Bartesaghi S Radi R Kinetic and mechanistic considerations to assess the biological fate of peroxynitrite. Biochim Biophys Acta (2014) 1840(2):768–80. 10.1016/j.bbagen.2013.07.005 PMC3858447 23872352

[48] Lancaster JR Jr. Nitric oxide: a brief overview of chemical and physical properties relevant to therapeutic applications. Future Sci OA (2015) 1(1):FSO59. 10.4155/fso.15.59 28031866 PMC5137977

[49] Davies MJ Myeloperoxidase-derived oxidation: mechanisms of biological damage and its prevention. J Clin Biochem Nutr (2011) 48(1):8–19. 10.3164/jcbn.11-006FR 21297906 PMC3022070

[50] Shao B Heinecke JW Using tandem mass spectrometry to quantify site-specific chlorination and nitration of proteins: model system studies with high-density lipoprotein oxidized by myeloperoxidase. Methods Enzymol (2008) 440:33–63. 10.1016/S0076-6879(07)00803-8 18423210

[51] Rubbo H Radi R Protein and lipid nitration: role in redox signaling and injury. Biochim Biophys Acta (2008) 1780(11):1318–24. 10.1016/j.bbagen.2008.03.007 18395525

[52] Mata-Perez C Sanchez-Calvo B Padilla MN Begara-Morales JC Valderrama R Corpas FJ Nitro-fatty acids in plant signaling: New key mediators of nitric oxide metabolism. Redox Biol (2017) 11:554–61. 10.1016/j.redox.2017.01.002 PMC5241575 28104576

[53] Deen AJ Sihvola V Harkonen J Patinen T Adinolfi S Levonen AL Regulation of stress signaling pathways by nitro-fatty acids. Nitric Oxide (2018)78:170–5. 10.1016/j.niox.2018.03.012 29567143

[54] Rubbo H Radi R Trujillo M Telleri R Kalyanaraman B Barnes S Nitric oxide regulation of superoxide and peroxynitrite-dependent lipid peroxidation. Formation of novel nitrogen-containing oxidized lipid derivatives. J Biol Chem (1994) 269(42):26066–75. 7929318

[55] O’Donnell VB Eiserich JP Chumley PH Jablonsky MJ Krishna NR Kirk M Nitration of unsaturated fatty acids by nitric oxide-derived reactive nitrogen species peroxynitrite, nitrous acid, nitrogen dioxide, and nitronium ion. Chem Res Toxicol (1999) 12(1):83–92. 10.1021/tx980207u 9894022

[56] Rubbo H Trostchansky A O’Donnell VB Peroxynitrite-mediated lipid oxidation and nitration: mechanisms and consequences. Arch Biochem Biophys (2009) 484(2):167–72. 10.1016/j.abb.2008.11.007 19022215

[57] Neves B Domingues P Oliveira MM Domingues MDR Melo T Profile of Phosphatidylserine Modifications under Nitroxidative Stress Conditions Using a Liquid Chromatography-Mass Spectrometry Based Approach. Molecules (2018) 24(1)107. 10.3390/molecules24010107 PMC6337642 30597957

[58] Montero-Bullon JF Melo T Rosario MDM Domingues P Liquid chromatography/tandem mass spectrometry characterization of nitroso, nitrated and nitroxidized cardiolipin products. Free Radic Biol Med (2019) 144:183–91. 10.1016/j.freeradbiomed.2019.05.009 31095999

[59] Melo T Domingues P Ribeiro-Rodrigues TM Girao H Segundo MA Domingues MRM Characterization of phospholipid nitroxidation by LC-MS in biomimetic models and in H9c2 Myoblast using a lipidomic approach. Free Radic Biol Med (2017) 106:219–27. 10.1016/j.freeradbiomed.2017.02.033 28219782

[60] Tsikas D Zoerner AA Jordan J Oxidized and nitrated oleic acid in biological systems: analysis by GC-MS/MS and LC-MS/MS, and biological significance. Biochim Biophys Acta (2011) 1811(11):694–705. 10.1016/j.bbalip.2011.06.015 21771665

[61] Senthilmohan R Kettle AJ Bromination and chlorination reactions of myeloperoxidase at physiological concentrations of bromide and chloride. Arch Biochem Biophys (2006) 445(2):235–44. 10.1016/j.abb.2005.07.005 16125131

[62] van Dalen CJ Whitehouse MW Winterbourn CC Kettle AJ Thiocyanate and chloride as competing substrates for myeloperoxidase. Biochem J (1997) 327(Pt 2):487–92. 10.1042/bj3270487 PMC1218820 9359420

[63] Franco-Pons N Casas J Fabrias G Gea-Sorli S de-Madaria E Gelpi E Fat necrosis generates proinflammatory halogenated lipids during acute pancreatitis. Ann Surg (2013) 257(5):943–51. 10.1097/SLA.0b013e318269d536 22964727

[64] de-Madaria E Molero X Bonjoch L Casas J Cardenas-Jaen K Montenegro A Oleic acid chlorohydrin, a new early biomarker for the prediction of acute pancreatitis severity in humans. Ann Intensive Care (2018) 8(1):1. 10.1186/s13613-017-0346-6 29330618 PMC5768584

[65] Albert CJ Crowley JR Hsu FF Thukkani AK Ford DA Reactive chlorinating species produced by myeloperoxidase target the vinyl ether bond of plasmalogens: identification of 2-chlorohexadecanal. J Biol Chem (2001) 276(26):23733–41. 10.1074/jbc.M101447200 11301330

[66] Thukkani AK Martinson BD Albert CJ Vogler GA Ford DA Neutrophil-mediated accumulation of 2-ClHDA during myocardial infarction: 2-ClHDA-mediated myocardial injury. Am J Physiol Heart Circ Physiol (2005) 288(6):H2955–64. 10.1152/ajpheart.00834.2004 15681699

[67] Meyer NJ Reilly JP Feng R Christie JD Hazen SL Albert CJ Myeloperoxidase-derived 2-chlorofatty acids contribute to human sepsis mortality via acute respiratory distress syndrome. JCI Insight (2017) 2(23)e96432. 10.1172/jci.insight.96432 PMC5752281 29212955

[68] Pike DP Vogel MJ McHowat J Mikuzis PA Schulte KA Ford DA 2-Chlorofatty acids are biomarkers of sepsis mortality and mediators of barrier dysfunction in rats. J Lipid Res (2020) 61(7):1115–27. 10.1194/jlr.RA120000829 PMC7328038 32376642

[69] Duerr MA Palladino END Hartman CL Lambert JA Franke JD Albert CJ Bromofatty aldehyde derived from bromine exposure and myeloperoxidase and eosinophil peroxidase modify GSH and protein. J Lipid Res (2018) 59(4):696–705. 10.1194/jlr.M083279 29444934 PMC5880502

[70] Heinecke JW Li W Mueller DM Bohrer A Turk J Cholesterol chlorohydrin synthesis by the myeloperoxidase-hydrogen peroxide-chloride system: potential markers for lipoproteins oxidatively damaged by phagocytes. Biochemistry (1994) 33(33):10127–36. 10.1021/bi00199a041 8060981

[71] Carr AC van den Berg JJ Winterbourn CC Chlorination of cholesterol in cell membranes by hypochlorous acid. Arch Biochem Biophys (1996) 332(1):63–9. 10.1006/abbi.1996.0317 8806710

[72] Hazen SL Hsu FF Duffin K Heinecke JW Molecular chlorine generated by the myeloperoxidase-hydrogen peroxide-chloride system of phagocytes converts low density lipoprotein cholesterol into a family of chlorinated sterols. J Biol Chem (1996) 271(38):23080–8. 10.1074/jbc.271.38.23080 8798498

[73] Sommerburg O Langhans CD Arnhold J Leichsenring M Salerno C Crifo C Beta-carotene cleavage products after oxidation mediated by hypochlorous acid–a model for neutrophil-derived degradation. Free Radic Biol Med (2003) 35(11):1480–90. 10.1016/j.freeradbiomed.2003.08.020 14642396

[74] Melo T Silva EM Simoes C Domingues P Domingues MR Photooxidation of glycated and non-glycated phosphatidylethanolamines monitored by mass spectrometry. J Mass Spectrom (2013) 48(1):68–78. 10.1002/jms.3129 23303749

[75] Simoes C Simoes V Reis A Domingues P Domingues MR Oxidation of glycated phosphatidylethanolamines: evidence of oxidation in glycated polar head identified by LC-MS/MS. Anal Bioanal Chem (2010) 397(6):2417–27. 10.1007/s00216-010-3825-2 20499053

[76] Bernoud-Hubac N Fay LB Armarnath V Guichardant M Bacot S Davies SS Covalent binding of isoketals to ethanolamine phospholipids. Free Radic Biol Med (2004) 37(10):1604–11. 10.1016/j.freeradbiomed.2004.07.031 15477011

[77] Sullivan CB Matafonova E Roberts LJ,2 Amarnath V Davies SS Isoketals form cytotoxic phosphatidylethanolamine adducts in cells. J Lipid Res (2010) 51(5):999–1009. 10.1194/jlr.M001040 19965577 PMC2853468

[78] Maciel E Faria R Santinha D Domingues MR Domingues P Evaluation of oxidation and glyco-oxidation of 1-palmitoyl-2-arachidonoyl-phosphatidylserine by LC-MS/MS. J Chromatogr B Analyt Technol BioMed Life Sci (2013) 929:76–83. 10.1016/j.jchromb.2013.04.009 23669606

[79] Maciel E Neves BM Santinha D Reis A Domingues P Teresa Cruz M Detection of phosphatidylserine with a modified polar head group in human keratinocytes exposed to the radical generator AAPH. Arch Biochem Biophys (2014) 548:38–45. 10.1016/j.abb.2014.02.002 24560783

[80] Flemmig J Spalteholz H Schubert K Meier S Arnhold J Modification of phosphatidylserine by hypochlorous acid. Chem Phys Lipids (2009) 161(1):44–50. 10.1016/j.chemphyslip.2009.06.144 19577554

[81] Egea J Fabregat I Frapart YM Ghezzi P Gorlach A Kietzmann T European contribution to the study of ROS: A summary of the findings and prospects for the future from the COST action BM1203 (EU-ROS). Redox Biol (2017) 13:94–162. 10.1016/j.redox.2017.05.007 28577489 PMC5458069

[82] Forman HJ Redox signaling: An evolution from free radicals to aging. Free Radic Biol Med (2016) 97:398–407. 10.1016/j.freeradbiomed.2016.07.003 27393004 PMC4996735

[83] Zheng Y Huang X Kelleher NL Epiproteomics: quantitative analysis of histone marks and codes by mass spectrometry. Curr Opin Chem Biol (2016) 33:142–50. 10.1016/j.cbpa.2016.06.007 PMC5129744 27371874

[84] Kaur S Baldi B Vuong J O’Donoghue SI Visualization and Analysis of Epiproteome Dynamics. J Mol Biol (2019) 431(8):1519–39. 10.1016/j.jmb.2019.01.044 30769119

[85] Domingues RM Domingues P Melo T Perez-Sala D Reis A Spickett CM Lipoxidation adducts with peptides and proteins: deleterious modifications or signaling mechanisms? J Proteomics (2013) 92:110–31. 10.1016/j.jprot.2013.06.004 23770299

[86] Stockwell BR Jiang X Gu W Emerging Mechanisms and Disease Relevance of Ferroptosis. Trends Cell Biol (2020) 30(6):478–90. 10.1016/j.tcb.2020.02.009 PMC7230071 32413317

[87] Ursini F Maiorino M Lipid peroxidation and ferroptosis: The role of GSH and GPx4. Free Radic Biol Med (2020) 152:175–85. 10.1016/j.freeradbiomed.2020.02.027 32165281

[88] Negre-Salvayre A Auge N Ayala V Basaga H Boada J Brenke R Pathological aspects of lipid peroxidation. Free Radic Res (2010) 44(10):1125–71. 10.3109/10715762.2010.498478 20836660

[89] Ni Z Fedorova M LipidLynxX: lipid annotations converter for large scale lipidomics and epilipidomics datasets. bioRxiv (2020). 10.1101/2020.04.09.033894 2020.04.09.033894.

